# Artificial Intelligence in Oral Cancer: A Comprehensive Scoping Review of Diagnostic and Prognostic Applications

**DOI:** 10.3390/diagnostics15030280

**Published:** 2025-01-24

**Authors:** Vineet Vinay, Praveen Jodalli, Mahesh S. Chavan, Chaitanya. S. Buddhikot, Alexander Maniangat Luke, Mohamed Saleh Hamad Ingafou, Rodolfo Reda, Ajinkya M. Pawar, Luca Testarelli

**Affiliations:** 1 Department of Public Health Dentistry, Manipal College of Dental Sciences Mangalore, Manipal Academy of Higher Education, Manipal 576104, Karnataka, India; vineet.mcodsmlr2023@learner.manipal.edu; 2 Department of Public Health Dentistry, Sinhgad Dental College & Hospital, Pune 411041, Maharashtra, India; 3 Department of Oral Medicine and Radiology, Sinhgad Dental College & Hospital, Pune 411041, Maharashtra, India; drmaheshschavan@gmail.com; 4 Department of Public Health Dentistry, Dr. D. Y. Patil Dental College and Hospital Pune, Dr. D. Y. Patil Vidyapeeth Pimpri Pune, Pune 411018, Maharashtra, India; chaitanya.buddhikot@dpu.edu.in; 5 Department of Clinical Science, College of Dentistry, Ajman University, Al-Jruf, Ajman P.O. Box 346, United Arab Emirates; a.luke@ajman.ac.ae (A.M.L.); m.ingagou@ajman.ac.ae (M.S.H.I.); 6 Centre of Medical and Bio-Allied Health Science Research, Ajman University, Al-Jruf, Ajman P.O. Box 346, United Arab Emirates; 7 Department of Oral and Maxillo-Facial Sciences, Sapienza University of Rome, Via Caserta 06, 00161 Rome, Italy; luca.testarelli@uniroma1.it; 8 Department of Conservative Dentistry and Endodontics, Nair Hospital Dental College, Mumbai 400034, Maharashtra, India

**Keywords:** artificial intelligence, convolutional neural network, dental, diagnosis, oral cancer, prognosis

## Abstract

**Background/Objectives**: Oral cancer, the sixth most common cancer worldwide, is linked to smoke, alcohol, and HPV. This scoping analysis summarized early-onset oral cancer diagnosis applications to address a gap. **Methods**: A scoping review identified, selected, and synthesized AI-based oral cancer diagnosis, screening, and prognosis literature. The review verified study quality and relevance using frameworks and inclusion criteria. A full search included keywords, MeSH phrases, and Pubmed. Oral cancer AI applications were tested through data extraction and synthesis. **Results**: AI outperforms traditional oral cancer screening, analysis, and prediction approaches. Medical pictures can be used to diagnose oral cancer with convolutional neural networks. Smartphone and AI-enabled telemedicine make screening affordable and accessible in resource-constrained areas. AI methods predict oral cancer risk using patient data. AI can also arrange treatment using histopathology images and address data heterogeneity, restricted longitudinal research, clinical practice inclusion, and ethical and legal difficulties. Future potential includes uniform standards, long-term investigations, ethical and regulatory frameworks, and healthcare professional training. **Conclusions**: AI may transform oral cancer diagnosis and treatment. It can develop early detection, risk modelling, imaging phenotypic change, and prognosis. AI approaches should be standardized, tested longitudinally, and ethical and practical issues related to real-world deployment should be addressed.

## 1. Introduction

Oral malignancies impact the oral cavity, including the mouth, tongue, lips, and throat. According to the World Health Organization (WHO), oral cancer is the sixth most common cancer worldwide, with more than 300,000 new cases identified each year [1]. The incidence of oral cancer varies significantly across global regions, with the highest rates reported in South and Southeast Asia.

Oral cancer exhibits higher prevalence in individuals who use tobacco products, consume excessive alcohol, and are infected with human papillomavirus (HPV). Oral cancer is more prevalent in men compared to women, and the likelihood of its occurrence increases with age. Prompt identification and timely intervention of oral cancer are crucial for enhancing outcomes. Prompt diagnosis and appropriate management of oral cancer result in a five-year relative survival rate of approximately 90% [2]. However, if cancer is diagnosed at an advanced stage, the five-year survival rate markedly decreases. The survival rate for oral cancer in India has improved due to timely detection and treatment [3]. Oral cancer (OC) can occur on the cheeks, lips, gum line, and posterior to the permanent teeth. Unhealing blisters or ulcers that produce discomfort or bleeding are cancer signs. Oral cancer (OC) symptoms include white or red lesions, non-healing sores on the lips, gum line, or tongue, a tumor in the oral cavity, compromised teeth, chewing or swallowing difficulty, speech difficulties, jaw pain, and a chronic sore throat. Leukoplakia is an early sign of oral cancer [4]. Oral cancer is common in India and has a higher fatality rate than in other nations.

The Indian Council of Medical Research reports that oral cancer is the most prevalent malignancy in men and the second in women. Approximately 10.3% of 2020 cancer cases were mouth cancer, according to GLOBOCAN 2020. The result was 75,290 deaths, ranking second. There were 300,413 instances over five years. Oral cancer is more common and more deadly in men [5]. Oral cancer is most often caused by tobacco usage, including smoking cigarettes, pipes, cigars, and smokeless tobacco products like chew and snuff. Tobacco use causes cancer worldwide, including mouth cancer [6]. Oral cancer is strongly linked to excessive alcohol use. It can independently raise oral cancer risk. When paired with smoke, the risk increases significantly. HPV infection is a risk factor for oral cancer, particularly oropharyngeal carcinoma, which affects the base of the tongue and tonsils. Poor dental care, wood smoking, and a diet low in fruits and vegetables can cause mouth cancer. These factors increase oral cancer risk [7].

Early detection of oral cancer is crucial; however, late detection generally leads to poor prognoses. The clinical presentation of oral cancer is unreliable as a predictor of its status, progression, or degree of dysplasia, making treatment choices inaccurate. The illness has numerous causes, and therapy is unexpected [8]. Researchers have developed numerous classifications for benign and malignant oral anomalies. The Apriori Algorithm has shown that all derived rules are highly confident, making them useful for oral cancer detection and prevention [9].

**Figure 1:**
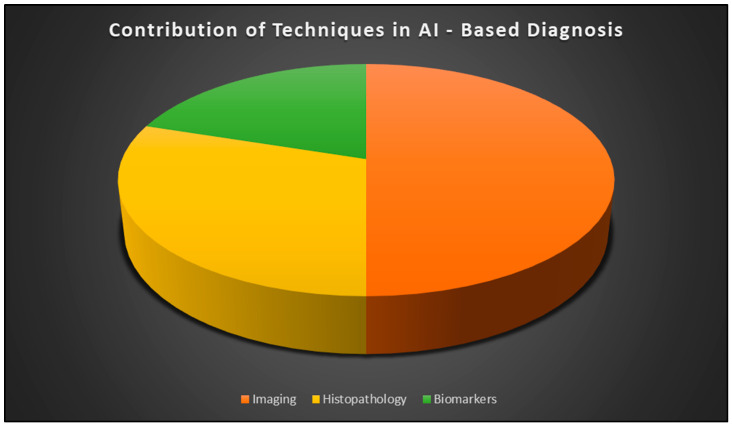
Contributions of major diagnostic techniques (imaging, histopathology, and biomarkers) to AI-based oral cancer research. Percentages represent the extent to which each technique supports advancements in diagnostic accuracy [10,11,12,13,14].

Oral lesions can be imaged using several methods. Literature suggests an image processor that can detect oral cancer using white light and autofluorescence [10]. More than two-thirds of oral lesions are found later in the disease, reducing survival probabilities [11]. Lesions are expensive to cure, especially in the late stages [12]. Medical professionals worry about delayed oral lesion diagnosis [13]. An automated system that can detect oral cancer without human interaction is needed to improve early detection and lessen the consequences of delayed diagnosis. Machine learning (ML) improves automated system classification precision. Deep learning (DL) can save humans from analyzing big datasets [14]. AI can help detect and predict oral cancer progression (Figure 1). It does this by reducing physicians’ load, complexity, and tiredness during diagnosis. Scientists around the world are interested in this technology because it mimics human cognition. This technology is new in dentistry, but the results are promising. Aubreville et al. [15] identified oral squamous cell cancer in confocal laser endomicroscopic images using deep learning methods. Additionally, Gupta et al. [16] used a deep architecture to diagnose dysplasia in microscopic images.

Recent technological advances are transforming healthcare. Due to increased use of emerging information and communication technologies, digital medicine is expected to change healthcare practices [17,18,19]. AI helps us make decisions by quickly analyzing and interpreting enormous amounts of data. Digital technologies benefit healthcare professionals, systems, and patients [19]. Recent technological advances are transforming healthcare. Due to increased use of ICT, digital medicine is expected to change healthcare professionals’ practices [17,18,19]. AI helps us make decisions by quickly analyzing and interpreting enormous amounts of data. Digital technologies benefit healthcare workers, systems, and patients [20].

This review seeks to comprehensively examine the use of artificial intelligence in diagnosing and predicting outcomes for oral cancer. It highlights the need to consolidate recent efforts aimed at early detection while addressing key challenges such as dataset limitations and ethical concerns. Additionally, the review identifies gaps in research to support the integration of AI into clinical practice.

- Discussion on the AI technologies implemented for oral cancer detection and diagnosis.
- The AI analysis of early discovery and its assessment result in an increase in diagnostic sensitivity.
- Using AI applications to predict oral cancer risk and prognosis.
- Identify the elements that are deficient and influence them towards areas of research that need to be probed in the future.

Detecting oral cancer at an early stage and making a rapid diagnosis are vital. The review will be an important resource for synthesizing the evidence on AI applications, and to identify possible routes of implementation from proof-of-concept stages, we lay down some foundations towards changes in patient outcomes. It also adds to the ability of AI in responding better than conventional methods for diagnosis. It heralds a new era of healthcare solutions that promise to treat oral cancer more efficiently in pain management [21]. Achieving these objectives would elucidate the potential of artificial intelligence in the early identification and management of oral cancer, hence enhancing healthcare delivery and outcomes.

## 2. Materials and Methods

### 2.1. Study Design

Prior to conducting the scoping review, the research question was formulated using the PCC Framework. This scoping review, has been registered on Figshare to ensure transparency and facilitate accessibility to the research materials. The Figshare repository includes the study protocol, detailed search strategies, inclusion and exclusion criteria, data extraction templates, and analysis framework. By registering this review, we aim to contribute to open science, encourage reproducibility, and provide a valuable resource for future research in this field. The review is available freely on Figshare (https://figshare.com/articles/preprint/Artificial_Intelligence_in_Oral_Cancer_A_Comprehensive_Scoping_Review_of_Diagnostic_and_Prognostic_Applications/26534305/1?file=48334843, 11 August 2024) [22]. We applied certain inclusion criteria to ensure that only relevant papers of adequate quality were included. The eligibility criteria for the studies were as follows. In accordance with the Arksey and O’Malley framework and the PRISMA-ScR (Preferred Reporting Items for Systematic Reviews and Meta-Analyses extension for Scoping Reviews) (see Supplementary Materials) standards, this study was carried out as a scoping review [23,24].

### 2.2. Data Collection

The data for this review were collected through a systematic search of PubMed and Google Scholar. The search strategy combined keywords and Medical Subject Headings (MeSH) terms related to artificial intelligence and oral cancer. The search terms included:

- Artificial Intelligence;
- Machine Learning;
- Deep Learning;
- Neural Networks;
- Oral Cancer;
- Oral Squamous Cell Carcinoma;
- Diagnosis;
- Screening;
- Prognosis;
- Imaging;
- Histopathology.

Search ((((((((((((((((Artificial Intelligence[Title]) OR Artificial Intelligence[Abstract]) OR Deep Neural Network[Title]) OR Deep Neural Network[Abstract]) OR Convolutional Neural Network[Title]) OR Convolutional Neural Network[Abstract]) OR Artificial Neural Network[Title]) OR Artificial Neural Network[Abstract]) OR AI[Title]) OR AI[Abstract])) OR Machine Learning[Title]) OR Machine Learning[Abstract])) AND ((((((((Mouth Neoplasm[Title]) OR Mouth Neoplasm[Abstract]) OR Oral Cancer[Title]) OR Oral Cancer[Abstract]) OR Oral Squamous Cell Carcinoma[Title]) OR Oral Squamous Cell Carcinoma[Abstract]) OR Oral Neoplasm[Title]) OR Oral Neoplasm[Abstract]))) AND ((((((((Diagnosis[Title]) OR Diagnosis[Abstract]) OR Prognosis[Title]) OR Progno-sis[Abstract]) OR Diagnostic Accuracy[Title]) OR Diagnostic Accuracy[Abstract]) OR Prognostic Accuracy[Title]) OR Prognostic Accuracy[Abstract]).

Publications from January 2000 to November 2024 were included in the search. The following were the requirements for inclusion:

- Population: Individuals diagnosed with oral cancer or at risk of its development.
- Interventions: The utilization of artificial intelligence, particularly machine learning methodologies such as deep neural networks (DNNs), has been associated with the detection of oral cancer and the screening of oral precancerous lesions.
- Results: Articles showcasing the precision, responsiveness, and selectivity, prognostic significance, and clinical outcomes of artificial intelligence (AI) implementations in oral cancer. The types of investigations include cohort studies, case–control studies, experimental clinical trials, and reviews/meta-analysis.

Non-English publications, studies lacking full-text access, and those unrelated to AI or oral cancer remained under the exclusion criteria.

### 2.3. Study Selection

Two reviewers (PJ and VV) independently examined the titles and abstracts of the identified papers to determine their applicability to the research issue. In accordance with the eligibility criteria, full-text versions of the relevant papers were obtained and further assessed for inclusion.

### 2.4. Data Extraction

A pretested, standardized data extraction form was used to extract data from the listed studies. Among the important data gathered were:

- Features of the study (author, year, and location);
- AI techniques used;
- Results that were reported, such as the sensitivity, specificity, and accuracy of the diagnosis.

### 2.5. Data Synthesis

Synthesized data were grouped topically to illustrate the evidence of AI applications in oral cancer diagnosis, screening, and prognosis. VV and PJ independently developed and separated concepts using an inductive method. The findings, which highlighted recurring themes and gaps in the literature, were presented both textually and tabularly.

## 3. Results

**Figure 2:**
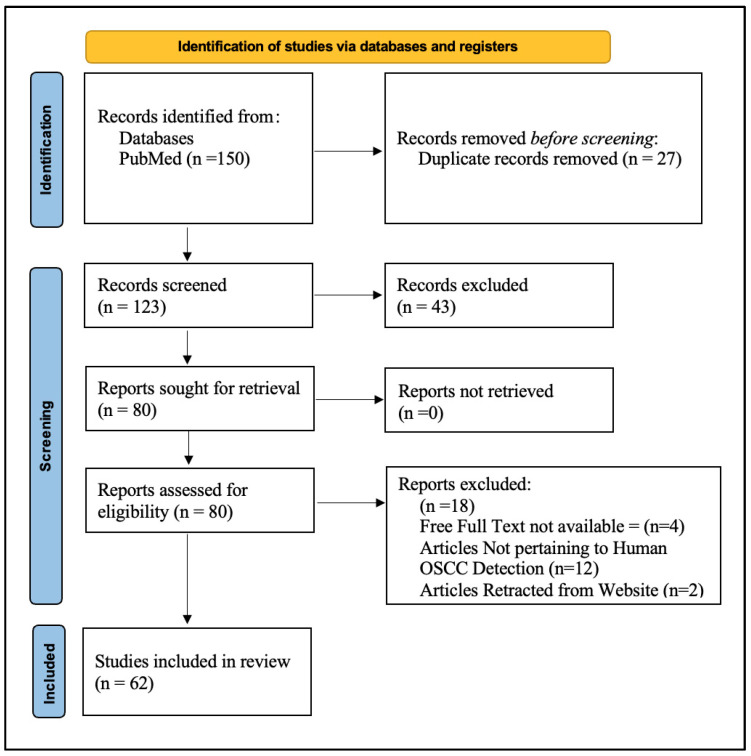
PRISMA 2020 flow diagram for segregating the studies to be included in the review.

Initially, our search strategy depicted 150 research articles. After 27 articles were found to be duplicated, a total of 123 records were initially screened by VV and PJ. A total of 43 records were excluded due to not pertaining to AI and OSCC. Finally, a total of 80 articles were sought for retrieval and assessed for eligibility criteria. Further, there were 18 articles which the authors strongly opined to exclude because of unavailability of free full text. Articles which did not pertain to Human OSCC and articles for which the abstract was present were further retracted from the database (Figure 2).

Finally, our overview of the studies included sixty-two studies (Table 1). A comprehensive evaluation was conducted with respect to the diverse AI technologies, including machine learning, deep learning, and artificial neural networks, in relation to various diagnostic tools and methodologies.

**Figure 3:**
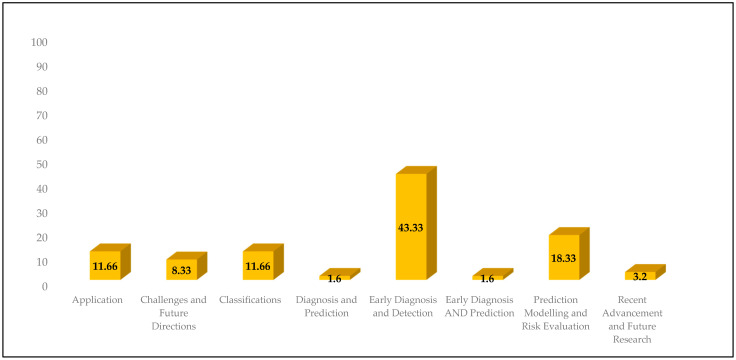
Themes generated from the existing evidence.

The themes derived from the existing literature are illustrated in Figure 3.

### 3.1. Applications

AI has demonstrated a great deal of promise in the detection of tumors in the mouth cavity and the oral and maxillofacial area.

1. Diagnostic Accuracy: AI can improve diagnostic accuracy and facilitate individualized treatment plans, according to Nishath Sayed Abdul et al. [25]. Despite its potential, acceptance for clinical usage is still heavily influenced by ethical and regulatory issues.
2. Datasets and Training: A challenge to implementing AI is the absence of reliable datasets. In order to support AI-assisted systems for diagnosing oral squamous cell carcinoma and other potentially malignant illnesses using machine and deep learning, Maria Clara Falcão Ribeiro-de-Assis et al. [26] presented the NDB-UFES dataset. Similar to this, Sara Bassani et al. [27] examined 13 studies that examined the detection of oral cancer and found that while AI algorithms showed promise, the majority used conventional histology. However, wider applicability was hampered by the scarcity of big training datasets.
3. Algorithm Performance: The effectiveness of various AI models has been the subject of numerous studies. Artificial neural networks (ANNs) fared noticeably better than conventional forecasting techniques in high-risk groups, according to Shruthi Hegde et al. [28]. However, after reviewing supervised machine learning techniques, Natheer Al-Rawi et al. [30] came to the conclusion that, when compared to other AI techniques, deep convolutional neural networks (DCNNs) were superior in early detection.
4. Sophisticated AI Models: For feature extraction and classification, Rana Alabdan et al. [31] presented a sophisticated model that combines DCNN and Inception modules. In terms of diagnostic accuracy, this model fared better than previous deep learning frameworks when combined with the moth flame optimization (MFO) technique.
5. Special Considerations: Anjali Pillai et al. [29] could not find any significant variations in survival rates between patient groupings; nevertheless, because of possible immunological dysregulation, patients with autoimmune conditions had somewhat lower survival rates. This emphasizes how crucial it is to take patient-specific aspects into account when implementing AI-driven solutions in healthcare settings.

### 3.2. Challenges

Shriniket Dixit et al. [32] have reviewed the possibility of early detection and treatment of oral cancer using AI. This review has pointed out the way in which AI, especially deep learning models, can perform analysis on large datasets obtained from the imaging modalities, thereby reducing some of the challenges associated with early-stage cancerous lesions. Further, the study also discusses risk reduction strategies and prevention of oral diseases. Shan Wang et al. [33] discussed studies on liquid biopsy biomarkers related to early detection in OSCC, including microbiome components, noncoding RNAs, extracellular vesicles, and ctDNA. The review encapsulates various screening techniques for OSCC and presents novel diagnostic options for the condition. A review by Kshreeraja Satish et al. [34] was performed regarding the implementation of omics data and machine learning techniques in unraveling the complexities of oral cancer. From a discussion on early detection, biomarker discovery, therapeutic targets, drug resistance, precision medicine, and prognostic biomarkers, an overview of current research being pursued in oral cancer using omics, bioinformatics, and machine learning is obtained. Yee-Fun Suet al. [35] review the current diagnostic methods for oral cancer and discuss possible future technologies aimed at improving diagnosis and treatment. The focus of the paper is on the expansion of diagnostic techniques in discussing possible improvement in the detection and treatment of oral cancerous lesions. Neda Haj-Hosseini et al. [36] discussed several oral cavity screening technologies, ranging from sample-based to direct screening techniques. This review widens the role of digitalization and automated analysis based on artificial intelligence, making these technologies more accessible and less dependent on highly trained specialists.

### 3.3. Classifications

Deepavalli Arumuganainar et al. [37] reported that the extra tree classifier predicted the interactomic hub genes with 98% accuracy and with 97% class accuracy. They identified HSPB1 as a hub gene using Cytohubba and hence proposed this classifier as potentially useful in the diagnosis and development of therapeutic strategies for oral cancer. Shintaro Sukegawa et al. [38] reported that oral pathologists with supplementary diagnoses obtained using deep learning models increased their diagnostic performance significantly. This hence shows the potential for involving reliable deep learning models in oral pathology diagnostics in improving diagnostic performance. Barnaby G. Ellis et al. [39] indicated that MLA succeeded in the detection of seven types of tissues present within the complex primary OSCC tumors, hence distinguishing three epithelial and four non-epithelial tissue types with high sensitivities and specificities. In this study, the sample size used for the study was small, but the findings will lead to future investigations on larger samples. Ji-Sun Kim et al. [40] reported an exceptionally high DOR of 121.66 for AI-assisted screening in detecting precancerous oral lesions. The subgroup analysis performed by them showed that OCT had more validity and a higher negative predictive value compared to photographic images and autofluorescence; hence, it is suggested that AI can be used as a non-invasive method for the early stages of diagnosis. Bo Fan Song et al. [41] presented a comparison of two transformer architectures, namely the Vision Transformer and Swin Transformer, applied in oral cancer image classification. Their experiment demonstrated that, with the pre-trained Swin Transformer architecture, they gained an accuracy of 88.7%, outperforming other deep learning models like ViT, VGG19, and ResNet50; it was shown that transformer-based architectures have great potential for advancement in oral image examination. Bonney Lee James et al. [42] examined the utilization of an automated image processing technique to differentiate between dysplastic-OPML and malignant lesions through OCT images. They also investigated methods in artificial neural networks to detect high-grade dysplasia. This study indicates that OCT may be particularly beneficial for the screening and monitoring of oral cancer, especially in resource-limited developing nations. Marc Aubreville et al. [15] suggested a novel methodology for the recognition of CLE pictures of oral cavity lesions, demonstrating superior performance compared to existing state-of-the-art techniques. The model attained an AUC of 0.96, a mean accuracy of 88.3%, and demonstrated great sensitivity and specificity, utilizing 7894 pictures of patients diagnosed with OSCC.

### 3.4. Diagnosis and Prediction

AI models have shown promising results in diagnosing oral cancer, distinguishing between normal and malignant areas, predicting patient survival, and grading the severity of the disease. These models have achieved high levels of accuracy, sensitivity, and specificity, outperforming traditional clinical methods. The use of AI in pathology can greatly enhance diagnostic outcomes and reduce errors. Regulatory bodies and policymakers should prioritize the approval and marketing of these AI products for clinical use [44].

### 3.5. Early Diagnosis and Detection

**Figure 4:**
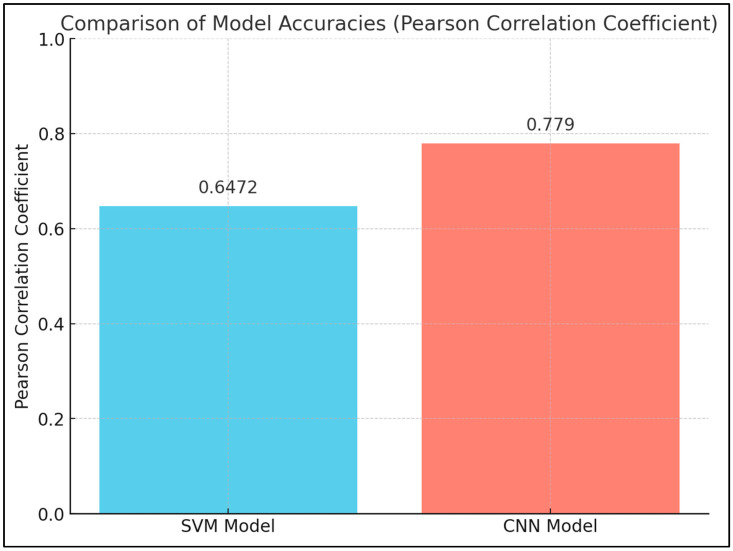
Comparison of accuracy across three commonly used AI models—Support Vector Machines (SVM) and Convolutional Neural Networks (CNNs)—highlighting their diagnostic capabilities in oral cancer detection.

Shubhangi Mhaske et al. [45] observed nuclear size, shape, and variation in chromatin distribution in both study and control groups. From the research, it can be identified that in their work, the Pearson correlation coefficient for the model SVM was 0.6472, while in the CNN model, it is 0.7790; hence, the latter model—which is CNN—is more accurate. The availability of multi-dimensional data and advances in AI give hope for better outcomes in oncology with respect to early-stage diagnosis and results of the patients’ outcomes (Figure 4). Examples are entropy and contrast among the textural features that are found in higher numbers in OSCC than among controls.

According to Preethi N Sharma et al. [46] computer-aided analysis of texture features proved helpful for early detection at the performance rate of 88% accuracy, 91% sensitivity, and 81% specificity. The assessment of Shaimaa O. Zayed et al. [47] on the validity of Diagnosis Oral Diseases Software or DODS showed a correct diagnosis rate comparable to oral pathologists performing microscopic examinations. Indeed, it has shown a high reliability and was quite accurate for diagnosis but not as good as oral pathologists holding advanced degrees. Aradhana Soni et al. [48] reported that the EfficientNetB0 model outperforms state-of-the-art approaches concerning oral cancer diagnosis based on histopathological images. It showed comparatively high values of accuracy, sensitivity, specificity, precision, F1 score, MCC, and kappa and hence holds immense potential to improve early diagnosis-automated strategies for the treatment. Ann-Kristin Struckmeier et al. [49] presented the sensitivity and specificity of contrast-enhanced CT scans as 76.85% and 82.20%, respectively, regarding the detection of bone invasion among oral cancer patients. Although several artifacts hampered assessment, the study concluded that a combination of multiple diagnostic approaches along with the employment of AI may lead to an improvement in the prehistopathological detection of bone invasion. Huixin Dou et al. [50] found SEMA3C to be highly expressed in poor-prognosis TSCC and also play an important role in cell adhesion and regulation processes. Overexpression of SEMA3C was identified to promote proliferation, migration, and invasion of tumor cells; thus, it will provide a potential biomarker for diagnosis, prognosis, and personalized medicine in TSCC. Dinesh Y et al. [51] estimated a machine learning algorithm trained on 300 clinical intra-oral images to find a moderate agreement with the clinicians when tested on 60 new images. It gave a mean average precision of 25.4%, with specificity at 75% and sensitivity at 88.9%. The results thus indicate that the model is helpful in early detection for the presence of suspected lesions in dental patients. The authors presented a system [53]. In the light of minimum time consumption using histopathological images for OSCC diagnosis, high accuracies were obtained by using the SVM algorithm in association with DenseNet201 combined with GLCM and HOG and LBP features. This would give high precision, sensitivity, specificity, and F-1 score; hence, it is useful in early diagnosis. Zihan Yang et al. [54] trained three varieties of CNNs utilizing OCT radiographs and discovered that, relative to machine learning techniques, CNN-based methods had a high accuracy of 96.76%. The findings demonstrated that deep learning algorithms can effectively assist in the detection and diagnosis of oral cancer. The AI method proposed by Kaori Oya et al. [55] detected OSCC from histopathological images with a very high accuracy of 99.65%. In the Grad-CAM analysis, it was observed that AI has given attention to cellular and structural atypia, especially in the basal layer, which showed that AI was appropriate for detection of OSCC.

A deep neural network model developed by Xiaoshuai Xu et al. [56] outperformed radiologists, surgeons, and students in the exact detection of positive lymph nodes in OSCC patients by using contrast-enhanced CT scans. High clinical accuracy suggests its application in precise diagnosis and personalized treatment planning. Accordingly, the predictive model developed with age and autoantibody levels improved the predictive capability of high-risk cases of OSCC, a model possibly usable through an online calculator that may reduce morbidity and mortality rates by providing personalized diagnostic information. John Adeoye et al. [59] identified 1745 differentially methylated CpG sites and 105 DMRs to discriminate OSCC. A linear SVM model using 11 optimal DMRs showed perfect performance that suggested saliva samples could be employed for OSCC screening. Along these lines, Mohanad A. Deif et al. [60] explored the effect of Reinhard stain normalization on diagnostic performance in histopathological images for oral squamous cell carcinoma. The XGBoost classifier coupled with Inception V3 and BPSO features had the highest performance, really proving its efficiency in improving diagnostic efficiency among OSCC patients. Praveen Birur N. et al. [61] assessed the application of telediagnosis and point-of-care devices for the screening of oral cancer in low-resource settings. This mobile phone and cloud technology was highly sensitive in the detection of lesions, and hence useful in early detection in such setups.

Suliman Mohamed Fati et al. [62] proposed a hybrid system using CNN models with other algorithms for the diagnosis of OSCC. The proposed method achieves 99.1% accuracy along with high values of sensitivity, specificity, precision, and AUC, which promise improved diagnostic methods. Hayato Tomita et al. [63] conducted a comparative study of the diagnostic performances of a deep learning model against that of radiologists regarding the classification of cervical lymph nodes in OSCC patients. The DL model performed significantly better, with higher accuracy, which indicated its promise as a tool for diagnosing cervical lymph nodes.

The accuracy of a tele-cytology platform called Cellscope in detecting oral lesions was assessed by Sumsum Sunny et al. [64]. It showed performances comparable to conventional cytology. When coupled to an artificial neural network in a risk stratification model, sensitivity and overall accuracy were improved; thus, it may potentially be useful for early detection and screening for oral cancer. In this connection, Ross D. Uthoff et al. [65] have performed a presentation of a smartphone-based intraoral probe for both auto-fluorescence and polarized white light imaging, which allowed performance of remote diagnosis based on cloud-based technology with a neural network. Field testing of this tool showed high accuracy and, therefore, is considered a very promising tool for oral cancer screening in areas of high risk. Hao Zhang et al. [66] proposed the automatic extent determination of oral cancer using various CNNs in tandem with morphological edge detection. This scheme has worked considerably in the diagnosis of oral cancer and has established a foothold in automated diagnostic applications. Xinxuan Zhou et al. [67] investigated the oral microbiota and found bacterial species highly associated with oral squamous cell carcinoma. Using machine-learning methods, diagnostic accuracy as high as 0.95 was obtained for this study, which means that oral microbiota data may provide a non-invasive, low-cost way to diagnose OSCC. By using machine learning, Xiaowei Song et al. [68] identified OSCC from premalignant lesions versus normal conditions at an accuracy of 86.7%. This study concludes that the combination of CPSI-MS and machine learning may be a reliable technique for clinical diagnosis in OSCC.

### 3.6. Early Diagnosis and Prediction

The research identified AUNIP, a gene, as a potential biomarker for the diagnosis and prognosis of oral squamous cell carcinoma (OSCC). The expression of AUNIP escalated with the severity of OSCC, and its inhibition curtailed cancer cell proliferation [70].

### 3.7. Prediction Modelling and Risk Evaluation

Yutaka Nikkuni et al. [71] reported the performance of their predictive models with AUCs ranging from 0.71 to 0.84, suggesting that the PET radiomics analysis had the potential to be useful in the preoperative prediction of cancer grade. In their study, Cheng Deng et al. [72] reported that their AI models reached an AUC of 0.92, with high sensitivity and specificity, hence outperforming even experienced radiologists in the diagnosis of lymph node metastases in OSCC patients. This suggests that AI may become a very valuable assistant in clinical practice. Jakob Einhaus et al. [73] applied a machine learning pipeline to classify histological grades in OSCC with high accuracy. They found that three features, which include granulocyte MAPKAPK2 signaling, stromal CD4+ memory T cell size, and the distance of fibroblast from the tumor border, were associated with clinical outcome and thereby provided a framework for analyzing complex imaging data to discover prognostic biomarkers. Rashmi Siddalingappa et al. [74] demonstrated the k-fold method with superior performance compared to the hold-out method in cancer stage prediction using the MAE score of 0.015. Their study led to the conclusion that the older the patients are, and the more mutations they have, the higher their risk for short survival and advanced stages of cancer.

Xinyi Zhang et al. [75] found that high-risk OL patients had approximately a four-fold increased risk of progressing to OC when compared to low-risk patients, with significant differences in time-to-progression. It remained predictive even after adjusting for age, oral leukoplakia site, and dysplasia grading, and hence may be beneficial for the early detection and prevention of high-risk oral leukoplakia patients. Tanvi Singh et al. [76] try to expand the number of cases in oral cancer gene datasets and find important genes for GBC prognosis. They have applied both supervised and unsupervised machine learning methods in their research. They indicated that auto-identification of the GBC-related genes, as well as other types of oral cancer, is crucial. Li-yuan Zhang et al. [77] proposed a machine learning-based IRS model for predicting the overall survival and disease-free survival of the OSCC patients. This multi-gene model was more accurate when compared to the conventional indicators and thus clinically applicable in combination with other factors. It was also associated with the tumor microenvironment and could predict the responses of immunotherapy and chemotherapy. Yeongjoo Kim et al. [78] attained this performance on an ORCA dataset which was based on seven differentially expressed genes between the risk groups. The results showed that TILs play a critical role in the tumor microenvironment and thus provide great potential for deep-learning approaches in prognosis.

Seda Camalan et al. [79] applied a clinical photographic image analysis method on the UK and Brazil datasets with accuracies of 73.6% and 90.9%, respectively, using different validation methods. They demonstrated a better performance when the proposed method used image patches rather than the whole image and identified predictive regions using class activation map analysis. K. Hamana et al. [80] reported that more than 70% of the DNA samples from tumor tissues and serum showed abnormalities in at least one gene examined, although the patterns of abnormality for serum DNA agreed well with those for tumor DNA. Pre- and post-surgery abnormalities were frequently observed, while no patient without such abnormalities after surgery had recurrence, whereas those who continued to show abnormalities had an increased risk for distant metastasis and death. Therefore, this result implies that the status of microsatellites in serum DNA could have predictive value for disease prognosis.

Ruoyan Cao et al. [81] identified a 3-mRNA signature of CLEC3B, C6, and CLCN1 as a prognostic biomarker pattern in OSCC. The risk score developed from the three mRNA expression levels had good predictive accuracy at 3 and 5 years both in training and validation cohorts. The present investigation identified neuroactive ligand–receptor interaction as the most enriched route linked to OSCC and emphasized this 3-mRNA signature as a valuable instrument for predicting OSCC patient survival.

### 3.8. Recent Advancement and Future Research

**Figure 5:**
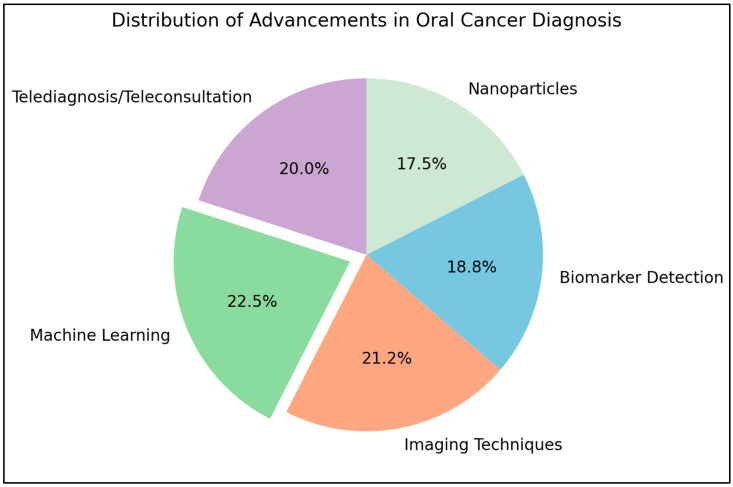
Pie chart illustrating the distribution of advancements in oral cancer diagnostic strategies (data adapted from the review by Rebeka Thiara Nascimento dos Santos et al. [83]).

Recent advancements in imaging techniques, including narrow band imaging, fluorescence imaging, and optical coherence tomography, have demonstrated potential for the early diagnosis of lesions in oral cancer (Figure 5). The review emphasized the potential for biomarker detection in saliva and the application of tailored nanoparticles for early diagnosis. The authors examined the importance of machine learning in enhancing diagnostic accuracy, while emphasizing the necessity for additional clinical and large-scale investigations to facilitate widespread application of the technologies [82]. Rebeka Thiara Nascimento dos Santos et al. [83] discussed telediagnosis and teleconsultations among other digital strategies to achieve a diagnosis of oral cancer based on clinical or histopathological images. The success of such strategies involves the amount of agreement between the digital diagnoses and those achieved conventionally. Better agreement reflects improved performance of such digital approaches.

## 4. Discussion

Resistance to waiting times and pathologist shortages have flourished based on the cases of AI integration pre-diagnosis in oral cancer burden. AI, especially through a variety of use cases around machine learning, deep learning and artificial neural networks (neural nets), has shown promise in enhancing the accuracy productivity and performance that comes with cancer detection as well as treatment planning.

AI has the potential to reduce healthcare inequities globally, particularly in areas where access to expert oncology care is limited. Its incorporation into smartphone-based probes and telemedicine systems may democratize the diagnosis and treatment of oral cancer, promoting fair access to high-quality care. To guarantee uniform standards and practices, international cooperation and policy harmonization are also required.

Diagnosis Accuracy—One of the most enormous contributions by AI in oral cancer is its ability to increase diagnostic accuracy. Classical diagnostic approaches predominantly involve visual inspection, histopathologic survey and expert opinions that may introduce human errors and variation. Background AI models, particularly convolutional neural networks (CNNs), have demonstrated excellent diagnostic accuracy in the context of medical images including but not limited to fluorescence imaging and exfoliative cystoscopy for diagnosing malignant and potentially malignant lesions with high sensitivity (García-Pola et al.) [24]. Therefore, by detecting architectural patterns from histochemical images as well of cytology samples, which is more consistently accurate than human experts, it may significantly reduce the number or oral cancer diagnostic errors [24].

The availability of non-invasive screening methods can equally assist in the early detection of disease subtype, therefore reinforcing AI’s imperative contribution to management. Especially against the backdrop of this pandemic, these AI-driven telemedicine platforms and smartphone-based probes are a cost-effective way to detect cancer early—especially in rural or underprivileged areas. For example, such diagnostic tools can rapidly analyze images taken by non-experts and provide timely referrals for interventions in places where specialist healthcare might be hard to reach. Ilhan et al. [17] also discussed matching AI-driven tools to minority patient populations in short supply of healthcare resources.

AI can predict rather than diagnose. AI is not all about prediction modelling and risk assessment. These AI models also significantly outperformed a lower-performing traditional statistical computational method (i.e., logistic regression, LR)—factoring in demographics and lifestyle data from patient questionnaires with biomarker results to predict the risk of progression to oral cancer (Alhazmi et al. [84]). Meanwhile, another study (2023) [85] showed that AI-based models might achieve higher precision than conventional methods in predicting cancer risk, which may potentially increase their importance and utility for surveillance screening of high-risk populations with selected preventive measures and alter the value proposition on reimbursement from physical resources/hooks/treatments to new data. This approach may enable improved predictions, not only allowing for early warning but also influencing individualized and pre-emptive health care.

Prognosis and treatment planning of oral cancer in addition to detection: AI has also been used in risk stratification prognosis prediction, as well as clinical decision making for the management of diseases [2]. These features are associated with such crucial clinical prognostic decision making for treatment responses and future outcomes that the AI systems of automated histopathological image analysis can be well suited to perform. AI can also be used to improve the accuracy of margin detection, where more work needs to be carried out, since more accurate margins are required for proper extraction of tumors and a reduced risk of recurrence. Kim et al. (2022) [40] also mentioned that the integration of AI with imaging technology, like Optical Coherence Tomography (OCT), can promise significant procedural enhancements and better treatment management insights. AI can help clinicians to imagine various treatment options and predict the effects of personalized treatments, which may lead not only to an improved chance for survival but also a better quality of life. Furthermore, the integration of AI into clinical workflows could redefine the role of clinicians, transitioning from manual diagnostics to oversight of AI-driven systems. This shift necessitates extensive training for healthcare professionals to interpret AI outputs effectively. By incorporating AI in real-time clinical decision making, practices can optimize treatment protocols and enhance patient outcomes. The success of such integration depends on multi-level collaboration between technologists, clinicians, and policymakers.

The application of artificial intelligence (AI) in the detection and diagnosis of oral cancer has garnered significant attention in recent years, with various studies highlighting its potential to enhance diagnostic accuracy and facilitate personalized treatment strategies. Nishath Sayed Abdul et al. (2023) [25] emphasized the role of AI in tumor detection within the oral cavity, suggesting that it could improve diagnostic precision and patient outcomes. However, ethical and regulatory challenges remain a concern in the integration of AI into clinical practice [26].

The introduction of the NDB-UFES dataset has been a pivotal development, as it aims to support research in AI-assisted systems for diagnosing oral potentially malignant disorders and oral squamous cell carcinoma (OSCC) using machine and deep learning techniques [26]. A review by Sara Bassani et al. [27] noted that while traditional histopathology remains the gold standard for diagnosing oral cancer, AI algorithms show promise, albeit hindered by the scarcity of large training datasets.

Several studies have demonstrated the efficacy of AI models in diagnosing oral cancer. Studies consistently highlight the superiority of advanced AI models like ANNs and DCNNs in early oral cancer detection. Notably, optimization techniques such as IDCNN paired with moth flame algorithms further enhance diagnostic accuracy [28,29,30,31]. Despite the advancements, challenges persist in the early detection and treatment of oral cancer. Key challenges include the need for robust datasets and advanced analytical methods. Emerging solutions like liquid biopsy biomarkers and the integration of omics data with machine learning offer promising avenues for early detection and complexity management [32,33].

The classification of oral cancer has also seen improvements through AI. Deepavalli Arumuganainar et al. [37] reported that the extra tree classifier achieved high accuracy in predicting interatomic hub genes, while Shintaro Sukegawa et al. [38] noted that deep learning models significantly enhanced the diagnostic performance of oral pathologists. Furthermore, Barnaby G. Ellis et al. [39] demonstrated that machine learning algorithms could effectively differentiate between various tissue types in OSCC tumors.

AI’s role in diagnosis and prediction is increasingly recognized, with models achieving high accuracy in distinguishing between normal and malignant areas, predicting patient survival, and grading disease severity [69]. The integration of AI into pathology could significantly enhance diagnostic outcomes and reduce errors, prompting calls for regulatory bodies to prioritize the approval of AI products for clinical use.

Recent advancements in imaging techniques, such as narrow band imaging and optical coherence tomography, have shown promise for early diagnosis of oral cancer lesions. The potential for biomarker detection in saliva and the use of tailored nanoparticles for diagnosis have also been highlighted [83]. However, the authors stress the need for further clinical investigations to facilitate the widespread application of these technologies.

Although impressive developments have been made, there are still many obstacles and constraints in the improvement of AI implementations availing oral cancer management. Heterogeneity in the data and lack of uniformity between studies is a major difficulty which puts constraints on generalizability, scalability, reproducibility and reliability of AI models across clinical practice (García-Pola et al.) [24]. They also pointed out that for the improvement in generalization over diverse populations and clinical settings, standardization is a crucial requirement.

The availability of long-term longitudinal studies to assess the clinical efficacy and overall survival associated with AI is another limitation (Sawhney et al.) [86]. We believe that research on the impact of AI should be extended to a period longer than 12 months and across demographically diverse patients. And while AI holds considerable promise for transforming how we predict and treat disease in the clinical setting, its practical integration will take time; it is not consumer-ready yet. The absence of this kind of integration may impede the wide adoption and implementation of AI technologies in clinical settings.

In addition, a general concern to be addressed is the ethical and legal side of AI applied in healthcare; specifically, fundamental inquiries into patient data protectionism necessitate context-aware solutions for more transparent algorithms with end-result accountability [87]. The authors stressed the need for creating inclusive ethical guidelines and regulatory infrastructure to mitigate these issues, thus enabling a safe and fair implementation of AI in the future prospects of oral cancer diagnostics.

One of the main hurdles in applying AI to oral cancer diagnostics is the lack of sufficient and diverse datasets. Many datasets are either too limited in size or fail to represent a broad spectrum of populations, making it difficult to create AI models that perform consistently across different demographics and clinical settings [88]. Moreover, the absence of standardized practices for collecting and annotating data introduces inconsistencies, which can negatively impact the reliability and accuracy of AI systems [89]. These challenges hinder the development and clinical implementation of robust AI tools. Addressing this issue requires collaboration to establish larger, more diverse, and standardized datasets, which would significantly enhance the effectiveness and applicability of AI in oral cancer care.

**Figure 6:**
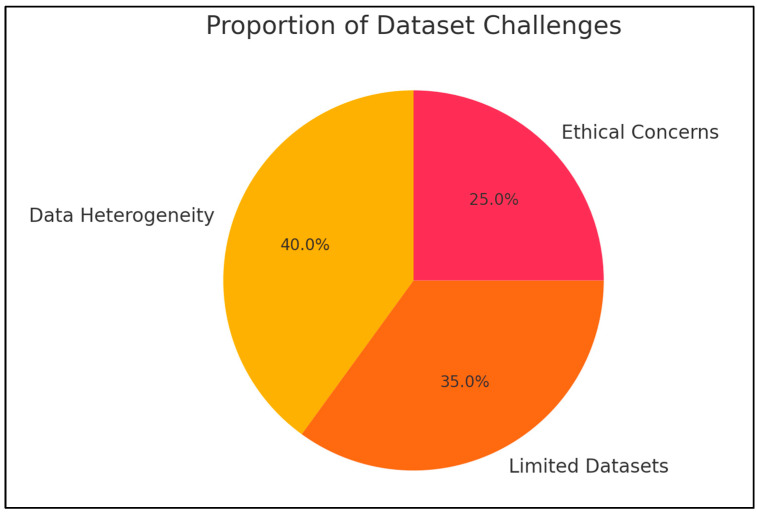
Distribution of key challenges associated with datasets in AI-based oral cancer research, including data heterogeneity, limited datasets, and ethical concerns. Percentages represent the proportion of studies identifying these challenges.

A significant hurdle in the application of artificial intelligence (AI) for diagnosing and predicting outcomes in oral cancer is the diversity of data. Current datasets often lack sufficient size and representation across various populations, which complicates the ability to generalize AI models in different demographic and clinical contexts [88,89]. For example, many datasets are confined to specific regions, limiting the effectiveness of AI systems for a broader range of patients. Furthermore, variations in data collection methods, standards for annotation, and imaging techniques worsen this issue, leading to decreased reliability and reproducibility of AI results (Figure 6).

To overcome these challenges, it is essential to create standardized datasets and protocols. Collaborative efforts among multiple institutions can help generate extensive and varied datasets that encompass different demographics, imaging techniques, and clinical scenarios. These datasets should follow standardized practices for data collection and annotation to enhance consistency and improve the reliability of AI models. Additionally, integrating longitudinal data can offer insights into disease progression and enhance the predictive power of AI systems [90].

The integration of AI tools into clinical practice also faces significant obstacles. Many AI models are developed in controlled environments that may not reflect the variability found in real-world clinical settings. For effective integration, AI systems must undergo testing across a variety of clinical scenarios and be validated against established diagnostic methods. Training programs for healthcare professionals are crucial to ensure these technologies are utilized effectively. Such programs should emphasize understanding AI outputs, identifying potential biases, and seamlessly incorporating AI tools into existing workflows [91].

To ensure responsible AI deployment, policymakers must create guidelines that address the ethical challenges of patient privacy, algorithmic transparency, and bias mitigation. For instance, regulatory frameworks could mandate the validation of AI systems in diverse clinical environments to improve reliability and fairness. Additionally, incentivizing the development of interoperable AI tools could enable seamless integration into existing healthcare infrastructures globally.

Introducing AI into the diagnosis and treatment of oral cancer brings with it several ethical concerns [92]. Ensuring the privacy of patient information and maintaining secure data handling are of utmost importance. Additionally, biases in AI systems—often resulting from incomplete or unrepresentative datasets—can lead to disparities in outcomes, potentially disadvantaging certain patient groups. The absence of clear regulatory frameworks further complicates the ethical integration of AI in healthcare [93]. To mitigate these issues, there is a pressing need for comprehensive ethical standards, transparent algorithmic accountability, and equitable access to AI technologies. By addressing these factors, the healthcare field can ensure that AI is integrated responsibly and fairly into oral cancer management.

The ethical dimensions of employing AI in oral cancer care are critical. Protecting patient privacy and data security is paramount, especially since AI models often require large datasets that may contain sensitive information. The potential for data breaches or misuse poses a significant barrier to widespread adoption. Moreover, biases inherent in AI systems—arising from incomplete or unrepresentative datasets—can result in inequities in healthcare outcomes that disadvantage certain patient groups [93].

To address these ethical concerns, robust frameworks for data governance must be established. These frameworks should include clear protocols for data anonymization, secure storage solutions, and controlled access measures. Institutions should adopt advanced encryption methods and conduct regular audits of their data management practices to ensure compliance with privacy regulations.

Our findings were in consensus with the article published by Kováč P et al. [94], wherein the authors examine AI-based phenotyping in the EU, emphasizing its potential to revolutionize public health while addressing the legal and ethical challenges it presents. It stresses the importance of balancing innovation with the protection of individual privacy rights. The authors suggested and highlighted the necessity for robust safeguards to ensure that the use of AI in healthcare, including forensic applications, does not compromise individual rights. Additionally, the paper points out the critical intersection of AI technology and sensitive genetic data, which requires proactive cybersecurity measures to protect against potential threats. It calls for current and future efforts to focus on securing AI models from attacks, maintaining data integrity.

We have also found that our findings were in consensus with Kharche et al. [95], who suggested that challenges remain, including data privacy concerns, the need for high-quality training datasets, and the integration of AI into existing healthcare systems. The future of AI in oral cancer diagnosis will depend on improving algorithm accuracy, ensuring equitable access, and fostering collaboration among technologists and healthcare providers. Overall, AI has the potential to revolutionize oral cancer diagnostics, making it more accessible and efficient, particularly in resource-limited settings.

Additionally, it is vital to develop transparent AI algorithms that provide interpretable outputs. This transparency allows clinicians to comprehend the rationale behind AI-driven decisions, fostering trust among healthcare providers and patients alike. Regulatory bodies should actively establish ethical standards for AI usage in healthcare to ensure accountability and equitable access to these technologies.

Overall, we found that our results were in consensus with Kavuluri et al. [96]. Despite these advancements, the article notes challenges in widespread adoption, including the need for specialized training, ethical considerations regarding patient privacy, and the necessity for robust data protection measures. The authors advocate for interdisciplinary collaboration among healthcare professionals and the establishment of guidelines to ensure effective implementation. Ultimately, the review underscores AI’s potential to revolutionize oral cancer diagnostics, making early detection more accessible and efficient, thereby improving overall healthcare delivery.

By addressing these challenges comprehensively, AI has the potential to transform the diagnosis, treatment, and management of oral cancer, ultimately enhancing patient outcomes and advancing oncology as a field.

## 5. Conclusions

To summarize, the incorporation of artificial intelligence into the diagnosis and treatment of oral cancer marks a significant breakthrough in healthcare innovation. AI’s capacity to facilitate early detection, refine risk evaluation, and optimize prognostic assessments holds immense promise for improving patient outcomes and transforming clinical practices. Nonetheless, realizing this potential requires addressing key challenges such as ensuring data diversity, navigating ethical concerns, and establishing standardized protocols. Future efforts should prioritize long-term studies that not only validate AI systems but also investigate their applicability across varied clinical environments. Encouraging collaboration among scientists, healthcare providers, and regulatory organizations will be crucial in advancing cutting-edge AI solutions that enhance the accuracy and efficiency of oral cancer care. Ultimately, adopting these sophisticated technologies will elevate diagnostic precision and usher in a new age of personalized oncology.

## Figures and Tables

**Table 1:** Summary of themes and related evidence of the included studies.

Sr. No	Themes Generated	Analysis Performed Using	Authors	Outcomes Reported
1	Application	Pathology	Abdul NS [25]	Artificial intelligence has proven beneficial in identifying tumors of the mouth cavity and forecasting the progression of oral and maxillofacial pathology. It can precisely identify people at elevated risk for oral cancer and the probability of disease recurrence.
2	Application	Histopathological Images	Ribeiro-de-Assis MCF [26]	The NDB-UFES dataset has been released to assist researchers in creating AI tools for the automated identification of oral potentially malignant diseases and oral squamous cell carcinoma. This dataset is designed for application in the domain of Artificial Intelligence, particularly in machine learning and deep learning.
3	Application	Histopathological Images	Bassani S [27]	Artificial intelligence techniques exhibit potential for enhancing diagnosis; nevertheless, the absence of extensive training datasets presents a significant problem that must be resolved.
4	Application	-	Hegde S [28]	This article examined the applications and benefits of artificial intelligence in oral cancer screening, diagnosis, prediction, treatment planning, and prognosis. The constraints and prospective advancements of AI in OC research were also examined.
6	Application	Immunological Assay	Pillai A [29]	The study revealed no significant differences in survival rates among groups; however, patients with autoimmune diseases exhibited marginally poorer results. This indicates that immunological dysregulation in these patients may influence their cancer outcomes.
7	Application	-	Al-Rawi N [30]	Supervised machine learning attained diverse degrees of accuracy, sensitivity, specificity, and AUC in the detection of oral cancer. No consensus exists about the optimal AI strategy for detection; however, deep learning, particularly deep convolutional neural networks, showed superior performance in early detection relative to supervised machine learning.
8	Application	Clinical Images	AlabdanR [31]	The research advocates for the implementation of the IDCNN model, which integrates the Inception module with DCNN, for feature extraction and classification purposes. The MFO approach helps enhance classification performance. The experimental findings indicate that the OIDCNN-OPMDD model surpasses other deep learning models.
9	Challenges and Future Directions	-	Dixit S [32]	This review discusses the potential use of (AI) in early detection and treatment of (OC). AI can accurately analyze large datasets from imaging modalities, and deep learning (DL) models have shown promise in overcoming challenges associated with early cancerous lesions. The review follows recommended guidelines and also discusses methods for reducing risk factors and preventing oral diseases.
10	Challenges and Future Directions	Biomarkers	Wang S [33]	Investigations are underway about liquid biopsy biomarkers for the early identification of oral squamous cell carcinoma (OSCC). The biomarkers encompass microbiome constituents, noncoding RNAs, extracellular vesicles, and circulating tumor DNA. This study examines the screening techniques for OSCC and presents novel approaches for illness diagnosis.
11	Challenges and Future Directions	Biomarkers	Satish KS [34]	This paper examines the application of omics data and machine learning methodologies in tackling the intricacies of oral cancer. It encompasses subjects like early detection, biomarker identification, therapeutic targets, drug resistance, precision medicine, and prognostic biomarkers. This review seeks to summarize the contemporary research on oral cancer utilizing omics, bioinformatics, and machine learning methodologies.
12	Challenges and Future Directions	-	Su Y-F [35]	This article examines contemporary diagnostic techniques for oral cancer and investigates prospective future technologies for enhanced diagnosis and therapy. The objective is to broaden diagnostic alternatives and improve the capacity to accurately identify and manage oral malignant lesions.
13	Challenges and Future Directions	-	Haj-Hosseini N [36]	The article discusses various technologies used for oral cavity screening, including sample-based methods and direct screening techniques. It emphasizes the importance of digitalization and automated AI-based analysis in making these technologies more accessible and reducing the need for highly trained specialists.
14	Classifications	Genetics	ArumuganainarD [37]	The additional tree classifier precisely identified interactomic hub genes, achieving 98% overall accuracy and 97% class accuracy. HSPB1 was discovered as a key gene using Cytohubba analysis. This classifier may enhance diagnostic and therapeutic approaches for oral cancer.
15	Classifications	Histopathological Images	SukegawaS [38]	The study revealed that the diagnostic accuracy of oral pathologists was markedly enhanced by using the findings of a deep learning model as adjunctive diagnoses. This indicates that integrating deep learning models can improve the diagnostic efficacy of pathologists in oral pathology. This research underscores the efficacy of employing robust deep learning models in oral pathology diagnosis.
16	Classifications	FTIR Data	Ellis BG [39]	The MLA has demonstrated effectiveness in detecting seven tissue types in complicated primary OSCC tumors. It effectively distinguished among three epithelial and four non-epithelial tissue types with elevated sensitivities and specificities. The study employed a limited sample size; however, the results will guide further, more extensive research endeavors.
17	Classifications	Radiography, Clinical Images	Kim J-S [40]	AI-assisted screening for oral precancerous lesions demonstrated a diagnostic odds ratio (DOR) of 121.66. Subgroup study indicated that optical coherence tomography (OCT) had superior accuracy and negative predictive value relative to photographic pictures and autofluorescence. The detection of oral malignant lesions by AI may serve as a non-invasive and expedient diagnostic instrument for early identification.
18	Classifications	Clinical Images	Song B [41]	The study compares the effectiveness of two transformer architectures, Vision Transformer (ViT) and Swin Transformer, for oral cancer image classification. The pre-trained Swin Transformer achieved 88.7% accuracy, outperforming ViT, VGG19, and ResNet50 models. The results suggest that transformer-based architectures have potential for advancing oral cancer image analysis.
19	Classifications	Point of Care Device	James BL [42]	This research demonstrated that an automated image processing technique could precisely differentiate dysplastic-OPML from malignant lesions utilizing OCT images. They investigated the application of artificial neural network methodologies to detect high-grade dysplasia. The research indicates that OCT may serve as an effective instrument for the screening and monitoring of oral cancer in resource-limited environments.
21	Classifications	Laserendomicroscopy Images	Aubreville M [15]	A novel technique for identifying CLE pictures of oral cavity lesions surpasses existing state-of-the-art methods. The approach attained an area under the curve (AUC) of 0.96 and a mean accuracy of 88.3%, demonstrating elevated sensitivity and specificity. The research utilized 7894 pictures from individuals identified with (OSCC).
22	Diagnosis and Prediction	-	Khanagar SB [43]	Artificial intelligence models have demonstrated encouraging outcomes in the diagnosis of oral cancer, differentiating between normal and malignant regions, forecasting patient survival, and assessing disease severity. These models have attained elevated levels of accuracy, sensitivity, and specificity, surpassing conventional clinical techniques. The application of AI in pathology can significantly improve diagnosis results and minimize errors. Regulatory authorities and governments must prioritize the endorsement and commercialization of these AI solutions for clinical application.
23	Early Diagnosis and Detection	-	Li J [44]	AI-driven screening utilizing clinical photography has demonstrated exceptional diagnostic efficacy in identifying oral mucosal neoplastic lesions and oral potentially malignant disorders (OPMDs). It possesses elevated sensitivity, specificity, and negative predictive value. The implementation of AI in general practice can improve diagnostic proficiency without requiring costly imaging technology.
24	Early Diagnosis and Detection	Nuclear Parameters	Mhaske S [45]	The study found significant differences in nuclear size, shape, and chromatin distribution between the study and control groups. The SVM model had a Pearson correlation coefficient of 0.6472, while the CNN model had a coefficient of 0.7790, indicating that the SVM model was more accurate. The availability of multidimensional datasets and advancements in AI have led to increased use in oncology research, with potential improvements in early detection and patient outcomes.
25	Early Diagnosis and Detection	Color Intensity-Based Textural Features	Sharma PN [46]	The research indicated that some textural characteristics, including entropy and contrast, were elevated in oral squamous cell cancer (OSCC) relative to the control group. Analysis of the receiver operating characteristic curve indicated that the accuracy, sensitivity, and specificity of utilizing these features for diagnosis were 88%, 91%, and 81%, respectively. This indicates that computer-assisted textural analysis may be beneficial for the early diagnosis of oral cancer.
26	Early Diagnosis and Detection	Clinical-histopathological Images	Zayed SO [47]	The study found that the Diagnosis Oral Diseases Software (DODS) had a correct diagnosis rate comparable to oral pathologists using microscopic examination. The software had high reliability and accuracy, making it a useful diagnostic tool. However, it had lower accuracy, sensitivity, and specificity compared to oral pathologists with a master’s degree.
27	Early Diagnosis and Detection	Oral biopsy Histopathological images	Soni A [48]	The EfficientNetB0 model attained remarkable performance metrics, exceeding leading methodologies in the domain. It exhibited elevated accuracy, sensitivity, specificity, precision, F1 score, Matthew’s correlation coefficient (MCC), and kappa statistics. The model’s incorporation of deep learning methodologies demonstrates potential for the automated early diagnosis of oral cancer, enhancing treatment approaches.
28	Early Diagnosis and Detection	Radiographs (Contrast-enhanced CT)	Struckmeier A-K [49]	The research indicated that CT scans had a sensitivity of 76.85% and a specificity of 82.20% in identifying bone invasion in patients. The percentages of false positives and false negatives were 11.27% and 5.99%, respectively. Artefacts influenced the assessment in several individuals, excluding those with bone invasion. Factors including tumor size, depth of invasion, tumor localization, lymphatic invasion, and perineural invasion were linked to the erroneous identification of bone invasion. The research indicates that integrating several techniques and employing artificial intelligence or monitoring electrolyte imbalances may enhance the precision of bone invasion identification prior to histopathological examination.
29	Early Diagnosis and Detection	Genetic Analysis (SEMA3C)	Dou H [50]	SEMA3C, a gene associated with cell adhesion and regulation of various cellular activities, was found to be highly expressed in TSCC (tongue squamous cell carcinoma). High expression of SEMA3C was linked to poor prognosis in TSCC patients. Further analysis confirmed its expression in tumor cells and showed that its knockdown inhibited tumor cell proliferation, migration, and invasion. This study suggests that SEMA3C could serve as a biomarker for TSCC diagnosis, prevention, prognosis, and personalized medicine.
30	Early Diagnosis and Detection	Clinical Intraoral Images	Y D, Ramalingam [51]	The research employed a training dataset including 300 clinical photographs to instruct doctors and a machine learning system. A test dataset of 60 novel clinical photographs was subsequently assessed by both the physicians and the algorithm. The findings indicated a moderate concordance between the two, with a Mean Average Precision of 25.4%, precision of 29.8%, and recall of 32.9%. The machine learning model exhibited a specificity of 75% and a sensitivity of 88.9%. The study indicates that machine learning may serve as an effective instrument for the early identification of suspected lesions in dental patients.
31	Early Diagnosis and Detection	Radiographs (Contrast-enhanced CT)	Struckmeier A-K [52]	The study revealed that sensitivity was greater in the cohort with enlarged lymph nodes than in the cohort with atrophied lymph nodes. Nevertheless, specificity was greater in the cohort with liquefied lymph nodes. The false negative rate for emphasized lymph nodes was 13%, but the false positive rates varied from 8.82% to 51.80%. The diagnostic accuracy for identifying lymph node metastases was superior at levels IIa and IIb compared to level III. Patients exhibiting pronounced lymphadenopathy were more predisposed to possess a tiny, well-differentiated neoplasm. CT scans were deemed adequate for forecasting lymph node metastasis in individuals with oral squamous cell carcinoma, and the prospective incorporation of artificial intelligence and deep learning may enhance the accuracy of CT in identifying lymph node metastasis. Additional inquiry is required.
32	Early Diagnosis and Detection	Histopathological Images	Ahmad M [53]	The suggested system demonstrated encouraging outcomes for the rapid diagnosis of oral squamous cell cancer (OSCC) utilizing histology images. The support vector machine (SVM) algorithm, integrating DenseNet201 with GLCM, HOG, and LBP features, attained elevated accuracy, precision, sensitivity, specificity, and F-1 score.
33	Early Diagnosis and Detection	Radiographs (Optical Coherence)	Yang Z [54]	Three varieties of (CNNs) were developed and assessed utilizing four measures. The CNN-based methodologies were juxtaposed with machine learning techniques utilizing the identical dataset. The results indicated that CNNs achieved a superior classification accuracy of 96.76% in contrast to the machine-learning-based technique, which attained 92.52%. The model’s capacity to differentiate various oral tissues was assessed by visualizing lesions in OCT images. The research determined that deep learning algorithms can offer decision support for the efficient detection and diagnosis of oral cancer.
34	Early Diagnosis and Detection	Histopathological Images	Oya K [55]	The proposed method achieved a high accuracy of 99.65% with a specific input size. Grad-CAM analysis revealed that the AI focused on both cellular and structural atypia of SCC, particularly in the region surrounding the basal layer. Training AI with different magnification images simultaneously could be effective for diagnosing oral SCC.
35	Early Diagnosis and Detection	Radiographs (Contrast-enhanced CT)	Xu X [56]	A DNN model was constructed and achieved high accuracy in localizing lymph nodes and discriminating metastasis. The model performed better than radiologists, surgeons, and students in accurately identifying positive lymph nodes. The model’s clinical accuracy was also higher than that of the Radiology Department, suggesting its potential for accurate diagnosis and personalized treatment planning.
36	Early Diagnosis and Detection	Biomarkers	Jing F [57]	The research identified specific genes linked to the advancement of (OSCC). These genes effectively predicted the fate of OSCC patients, indicating that those in the high-risk category exhibited a poor prognosis. The seven identified significant genes may function as possible biomarkers for the prognosis of OSCC patients.
37	Early Diagnosis and Detection	Biomarkers	Tseng Y-J [58]	This study with 337 individuals showed that a predictive model incorporating age and autoantibody levels enhanced the predictive accuracy for high-risk instances of OSCC. The approach can be utilized professionally via an online calculator to deliver individualized information for OSCC diagnosis, potentially diminishing morbidity and fatality rates.
38	Early Diagnosis and Detection	Biomarkers	Adeoye J [59]	A study discovered 1745 differentially methylated regions (DMRs) and 105 differentially methylated cytosines (DMCs) for the detection of OSCC. The preponderance of DMRs exhibited hypermethylation, although the ratio of hypomethylated to hypermethylated DMCs was comparable. DMRs were predominantly annotated to promoter regions, whereas DMCs were primarily annotated to intergenic regions. A linear SVM model utilizing 11 optimum DMRs showed flawless efficacy in OSCC identification. Saliva samples can facilitate biomarker identification, and machine learning platforms may assist in the screening of OSCC.
39	Early Diagnosis and Detection	Histopathological Images	Deif MA [60]	The study examined the impact of Reinhard stain normalization on performance. The best features were extracted and selected, and XGBoost was used for classification. The highest accuracy of 96.3% was achieved with Inception V3 and BPSO. This approach improves diagnostic efficiency and reduces costs for OCSCC patients using histopathological images.
40	Early Diagnosis and Detection	Point of Care Device	Birur N P [61]	Onsite specialists assessed a small percentage of subjects and performed biopsies on some. The study found that telediagnosis had high accuracy compared to onsite specialists, while community health workers had lower sensitivity. The mobile phone and cloud technology accurately identified lesions, making it a useful tool for oral cancer screening in low-resource settings.
41	Early Diagnosis and Detection	Histopathological Images	Fati SM [62]	A technique employing hybrid features derived from CNN models and diverse algorithms attained exceptional outcomes in diagnosing the OSCC dataset. The dimensionality of the features was decreased by PCA before being input into an ANN algorithm, yielding encouraging accuracy. The proposed approach attained an accuracy of 99.1%, along with elevated specificity, sensitivity, precision, and AUC.
42	Early Diagnosis and Detection	Radiographs (Contrast-enhanced CT)	Tomita H [63]	The research evaluated the diagnostic efficacy of a deep learning (DL) model against radiologists’ evaluations in categorizing cervical lymph nodes (LNs) in individuals with OSCC. The deep learning model demonstrated markedly superior accuracy in differentiating between benign and metastatic lymph nodes relative to the evaluations of the radiologists. This indicates that deep learning analysis of pretreatment contrast-enhanced CT scans may serve as an effective instrument for diagnosing cervical lymph nodes in patients with oral squamous cell carcinoma.
43	Early Diagnosis and Detection	Point of Care Tele—Cytology	Sunny S [64]	The research assessed the precision of a tele-cytology platform named Cellscope in identifying oral lesions. The platform had an overall accuracy of 84-86% and exhibited comparable performance to traditional cytology. Nonetheless, it exhibited limited sensitivity in identifying specific lesions. The integration of image processing with an artificial neural network-based risk stratification model considerably enhanced sensitivity and overall accuracy. The research finds that tele-cytology, in conjunction with the risk stratification model, serves as an effective instrument for the early diagnosis and screening of oral cancer.
44	Early Diagnosis and Detection	Point of Care Device	Uthoff RD [65]	A remote specialist and a convolutional neural network (CNN) accurately identified 170 image pairs as ‘suspect’ or ‘not suspicious’, utilizing the on-site professional’s diagnosis as the reference standard. Sensitivities, specificities, and positive predictive values were reported.
45	Early Diagnosis and Detection	Clinical Images	Zhang H [66]	This research established a deep learning methodology to autonomously determine the extent of malignancy in oral photos. The approach utilized various forms of CNN and applied morphological edge detection for precise feature extraction. The experimental findings demonstrated that the approach was efficacious in diagnosing oral cancer.
46	Early Diagnosis and Detection	Microbial Analysis	Zhou X [67]	This study found that the average diagnostic accuracy rates for diagnosing oral squamous cell carcinoma (OSCC) using five different sites and saliva were 98.17% and 95.70%, respectively. Cross-validations showed estimated external prediction accuracies of 96.67% and 93.58%, respectively. The false-negative rate was 0%. The study also identified certain bacterial species that were strongly correlated with OSCC. Overall, the study suggests that using oral microbiota data and machine learning methods can provide a noninvasive and inexpensive method of diagnosis.
47	Early Diagnosis and Detection	Biomarkers	Song X [68]	Machine learning has been used to distinguish OSCC and premalignant lesions from normal conditions with an accuracy of 86.7%. This suggests that combining CPSI-MS and machine learning can be a reliable and automated tool for diagnosing OSCC in clinical settings.
48	Early Diagnosis and Detection	Point of Care Device	Uthoff RD [69]	A compact smartphone-based intraoral probe has been developed that allows for autofluorescence imaging and polarized white light imaging. It is small and flexible, allowing for imaging of high-risk areas for oral cancer. Remote diagnosis is possible through cloud-based technology and a neural network. Field-testing data are provided.
49	Early Diagnosis AND Prediction	Biomarkers	Yang Z [70]	The study found that AUNIP, a gene, could be a useful biomarker for diagnosing and predicting the prognosis of oral squamous cell carcinoma (OSCC). AUNIP’s expression increased with the severity of OSCC, and its suppression inhibited cancer cell growth. This suggests that targeting AUNIP could be a potential strategy for preventing and treating OSCC.
50	Prediction Modelling and Risk Evaluation	Histological Images	Nikkuni Y [71]	The models achieved areas under the curve ranging from 0.71 to 0.84, indicating the usefulness of PET radiomics analysis for preoperative prediction of the grade.
51	Prediction Modelling and Risk Evaluation	CT-MRI Radiographic Images	Deng C [72]	The AI models had an area under the curve (AUC) of 0.92 and high sensitivity and specificity. The performance of AI was better than that of experienced radiologists. This suggests that AI could be a useful tool in clinical practice for diagnosing LN metastases in OSCC patients.
52	Prediction Modelling and Risk Evaluation	Biomarkers	Einhaus J [73]	A machine learning process was employed to precisely diagnose the histological grade of OSCC. Three model parameters were identified as correlating with clinical outcomes: granulocyte MAPKAPK2 signaling, stromal CD4+ memory T cell size, and the distance of fibroblasts from the tumor margin. This study offers a framework for the analysis of intricate imaging data and the identification of prognostic biomarkers for recurrence risk assessment and the development of immunomodulatory therapies.
53	Prediction Modelling and Risk Evaluation	International Cancer Genome Consortium (ICGC) data portal.	Siddalingappa R, Kanagaraj S [74]	The experimental results indicate that the k-fold approach surpasses the hold-out method, achieving a mean absolute error score of 0.015. The model effectively categorizes patients into various stages of cancer, demonstrating good accuracy, recall, precision, and F-measure. The research suggests that elderly patients with a greater number of mutations have an elevated risk of reduced survival and advanced cancer stages.
54	Prediction Modelling and Risk Evaluation	Histopathological Images	Zhang X [75]	The study found that high-risk oral leukoplakia (OL) patients were almost 4 times more likely to develop oral cancer (OC) compared to low-risk patients. The time-to-progression also differed significantly between the two groups. The 5-year probability of OC development was higher in high-risk patients. The OMRS model remained predictive even after adjusting for age, OL site, and dysplasia grading. This model can help identify high-risk OL patients and improve early diagnosis and prevention of OC.
55	Prediction Modelling and Risk Evaluation	Genetics	Singh T [76]	The study aimed to increase the number of instances in oral cancer gene datasets and identify key genes for gingivobuccal cancer (GBC) prognosis. Supervised and unsupervised machine learning methods were used to annotate and analyze the datasets, emphasizing the significance of automated gene identification for GBC and other oral cancer types.
56	Prediction Modelling and Risk Evaluation	Genetics	Zhang L [77]	A study developed an IRS model using machine learning to predict the survival and disease-free survival of OSCC patients. The model included several genes and was found to be more accurate than traditional indicators. The model was also an independent risk factor and could be used in combination with other factors for clinical application. The IRS was associated with tumor microenvironment and could predict response to immunotherapy and chemotherapy. The study suggests that the model could help identify biomarkers and targets for immunotherapy.
57	Prediction Modelling and Risk Evaluation	Biomarkers	Kim Y [78]	A classifier was used to predict patient survival patterns using an ORCA dataset. Seven genes were identified as differentially expressed between risk groups. This study highlights the significance of TILs in the tumor microenvironment and suggests the potential of a deep learning approach for cancer prognosis.
58	Prediction Modelling and Risk Evaluation	Clinical Images	Camalan S [79]	A method for analyzing clinical photographic images was tested on datasets from the UK and Brazil. The system achieved accuracies of 73.6% and 90.9% using different validation methods. The study also found that using patches instead of the whole image improved performance and identified predictive regions using class activation map analysis.
59	Prediction Modelling and Risk Evaluation	Biomarkers	Hamana K [80]	A study found that a majority of DNA samples from tumor tissues and serum showed abnormalities in at least one gene. The patterns of abnormalities in serum DNA matched those found in tumor DNA. Abnormalities were often detected before and after surgery. Patients with no abnormalities after surgery had no recurrence and were disease-free, while those with abnormalities had a higher risk of distant metastasis and death. This suggests that analyzing microsatellite status in serum DNA could help predict disease prognosis.
60	Prediction Modelling and Risk Evaluation	Genetics	Cao R [81]	A 3-mRNA signature consisting of CLEC3B, C6, and CLCN1 was found to be a prognostic biomarker pattern for oral squamous cell carcinoma (OSCC). The risk score was calculated based on the expression levels of these mRNAs. The signature showed good predictive accuracy for 3- and 5-year survival in both the training and validation cohorts. Machine learning analysis also confirmed the significance of this signature in predicting survival. The pathway analysis revealed that neuroactive ligand–receptor interaction was the most enriched pathway associated with OSCC. Overall, the 3-mRNA signature is a promising tool for predicting OSCC patient survival.
61	Recent Advancement and Future Research	-	Ojha A [82]	This review discusses the advancements in imaging methods like narrow band imaging, fluorescence imaging, and optical coherence tomography for early lesion detection. It also highlights the use of biomarker detection in saliva and targeted nanoparticles for early diagnosis, along with the role of machine learning in improving diagnostic accuracy. However, further clinical and large-scale studies are needed for widespread adoption.
62	Recent Advancement and Future Research	-	Dos Santos RTN [83]	Digital strategies such as telediagnosis and teleconsultations are being used to diagnose oral cancer using clinical or histopathological images. The success of these strategies is determined by the level of agreement in the better performance of the strategy.

## Data Availability

All the data related to the study has been presented within the manuscript.

## Supplementary Materials

The following supporting information can be downloaded at: https://www.mdpi.com/article/10.3390/diagnostics15030280/s1, Preferred Reporting Items for Systematic reviews and Meta-Analyses extension for Scoping Reviews (PRISMA-ScR) Checklist. Reference [97] is cited in the Supplementary Materials.
